# The search for clarity regarding “clinically meaningful outcomes” in Alzheimer disease clinical trials: *CLARITY-AD and Beyond*

**DOI:** 10.1186/s13195-024-01412-z

**Published:** 2024-02-16

**Authors:** Rawan Tarawneh, Vernon S. Pankratz

**Affiliations:** 1 Department of Neurology and Center for Memory and Aging, University of New Mexico, Albuquerque, NM USA; 2 Department of Internal Medicine, Division of Epidemiology, Biostatistics, and Preventive Medicine, University of New Mexico, Albuquerque, NM USA

**Keywords:** Lecanemab, Alzheimer disease, Clinical outcomes, Clinically meaningful change, Minimal clinically important difference

## Abstract

CLARITY-AD is an 18-month, double-blinded, placebo-controlled, phase 3 trial which examined the safety and efficacy of the anti-amyloid agent, lecanemab, in mild cognitive impairment and mild dementia due to Alzheimer disease (AD). Lecanemab effectively reduced mean brain amyloid burden and was associated with statistically significant favorable effects, reflected by moderately less decline in the primary and secondary clinical outcomes, at 18 months compared to placebo. However, there is controversy within the AD community regarding the clinical significance of these results and whether they translate into clinically meaningful and tangible benefits on cognition or daily functions.

We here review the primary and secondary clinical outcomes of CLARITY-AD and present our interpretation of the potential clinical meaningfulness of the group-level differences in study outcomes in the context of the 18-month study duration. We propose that the validation of stage-appropriate group-level thresholds for clinical meaningfulness of AD trial outcomes in biologically confirmed cohorts will allow objective interpretation of trial results and guide clinical decision-making. Further, in accordance with FDA guidance which emphasizes patient-focused drug development, the contextualization of AD clinical trial outcomes can be facilitated by supplementary individual-level data analyses which measure the risk of disease progression or summarize intraindividual change, using prespecified thresholds of clinically meaningful change, in each of the study groups over the trial period. The concepts of “time-saved” and “time-based” slowing in disease progression can be used to communicate clinical outcomes associated with emerging disease-modifying AD therapies to various stakeholders. We also describe several factors that need to be considered when evaluating outcomes of emerging AD therapies, including disease stage, the neuropathologic complexity of AD, time-based effects of disease-modifying therapies, and the possible influence of individual factors on treatment response and/or risk for adverse events. The consideration of these factors in the design and reporting of future trials of emerging AD therapies will guide clinicians regarding their appropriateness for use in various patient populations.

Finally, we emphasize that data from clinical cohorts with longer durations of treatment and follow-up, including extension studies and patient registries, is needed to evaluate the long-term safety and efficacy of lecanemab in early symptomatic AD.

## Introduction

Lecanemab is a humanized IgG1 monoclonal antibody which selectively binds to soluble aggregated, and highly toxic, amyloid-β protofibrils [1]. In July 2023, lecanemab became the first disease-modifying Alzheimer disease (AD) treatment to receive full US Food and Drug Administration (FDA) approval, based on data from CLARITY-AD (ClinicalTrials.gov number, NCT03887455), an 18-month, multicenter, double-blinded, placebo-controlled, phase 3 trial which examined the safety and efficacy of lecanemab in individuals with mild cognitive impairment (MCI) or mild dementia due to AD [1, 2]. All participants in CLARITY-AD had biomarker evidence of brain amyloid pathology prior to randomization.

### CLARITY-AD: one step closer to effective disease modification

CLARITY-AD demonstrated effective reduction of mean brain amyloid burden on amyloid positron emission tomography (PET) imaging, and moderately less decline on primary and secondary clinical outcomes, at 18 months in the lecanemab arm compared to placebo [1]. Favorable effects of lecanemab were observed on the primary study endpoint, the mean change from baseline in the Clinical Dementia Rating-Sum of Boxes (CDR-SB) [3–5] scores at 18 months, and secondary clinical outcomes, including the mean change from baseline to 18 months in the 14-item cognitive subscale of the Alzheimer Disease Assessment Scale (ADAS-Cog14) [6], the Alzheimer Disease Composite Score (ADCOMS) [7], and the Alzheimer Disease Cooperative Study–Activities of Daily Living Scale for MCI (ADCS-MCI-ADL) [8], compared to placebo (*p* < 0.001) (Table 1)^1^. Lecanemab was also associated with beneficial effects on exploratory outcomes, including quality of life and caregiver burden [9, 10].

**Table 1:** Key findings from the CLARITY-AD study^a^

*Endpoint*^a^	*Adjusted least-squares mean change from baseline at 18 months*		*Adjusted mean group-level difference (95% CI)*	*p value*
	***Lecanemab***	***Placebo***		
***Primary efficacy endpoint***				
CDR-SB score	1.21	1.66	− 0.45 (− 0.67 to − 0.23)	*p* < 0.001
***Secondary efficacy endpoint***				
Amyloid burden on PET (centiloids)	− 55.48	3.64	− 59.12 (− 62.64 to − 55.60)	*p* < 0.001
ADAS-Cog14 score	4.14	5.58	− 1.44 (− 2.27 to − 0.61)	*p* < 0.001
ADCOMS score	0.164	0.214	− 0.050 (− 0.074 to − 0.027)	*p* < 0.001
ADCS-MCI-ADL score	− 3.5	− 5.5	2.0 (1.2 to 2.8)	*p* < 0.001

*CI* confidence interval, *CDR-SB* Clinical Dementia Rating-Sum of Boxes, *PET* positron emission tomography, *ADAS-Cog14* 14-item cognitive subscale of the Alzheimer Disease Assessment Scale, *ADCOMS* Alzheimer Disease Composite Score, *ADCS-MCI-ADL* Alzheimer Disease Cooperative Study-Activities of Daily Living Scale for Mild Cognitive Impairment

A total of *n* = 1734 participants were included in the primary endpoint analyses. Secondary endpoint analyses included *n* = 698, 1726, 1732, and 1579 participants for amyloid PET, ADAS-Cog14, ADCOMS, and ADCS-MCI-ADL, respectively

^a^The presence of brain amyloid pathology was confirmed by amyloid positron emission tomography (PET) scans or cerebrospinal fluid (CSF) testing in all participants prior to randomization

To many in the AD field, these are exciting results. As effective treatments for AD are limited, results of CLARITY-AD, and the subsequent full FDA approval of lecanemab, offer hope that we have come closer to disease-modifying treatments that are associated with favorable effects on clinical disease progression and are available to our clinic populations.

### Statistical vs clinical significance

Despite general enthusiasm for the positive results of CLARITY-AD, there is controversy within the AD community whether these statistically significant outcomes are “clinically significant” [11]. In other words, do they translate into “clinically meaningful” benefits that are likely to have a direct and relevant impact on patients and caregivers through “tangible” changes in cognition or daily functions? There is uncertainty among health care providers regarding the clinical interpretation of these findings, and what they really mean for patients and their families. Further, how can these results be best explained to patients and caregivers, and effectively translated into clear and well-informed decision-making conversations in the clinic?

Defining the “clinical meaningfulness” of trial outcomes for emerging AD therapies is challenging and often controversial. With varying opinions in the field, a simple and straightforward method to measure clinical meaningfulness has not yet been established. However, most agree that several factors need to be considered when evaluating clinical meaningfulness of AD trial outcomes: (i) an adequate understanding of the specific outcome measures and what they mean in terms of functional benefits or impact on daily activities, (ii) the extent to which clinical outcomes reported as group-level differences translate into discernible effects at the individual level, (iii) how patient and caregiver perspectives can be systematically integrated into definitions of clinical meaningfulness, (iv) the time needed to achieve a response, (v) estimating the projected benefit over time for disease-modifying effects during treatment and after drug discontinuation, (vi) the influence of disease stage and individual factors on response to treatment, and (vii) the inherent neuropathologic complexity of AD.

The purpose of this manuscript is to review published results from the CLARITY-AD phase 3 trial of lecanemab in early symptomatic AD [1] and discuss our interpretation of the potential clinical meaningfulness of the reported group-level differences in the primary and secondary clinical outcomes in the context of the 18-month trial duration. Importantly, we propose methods to facilitate objective interpretation of trial results and their translation into more easily communicable terms to patients and families. We also discuss several factors that may potentially influence our interpretation of the clinical relevance of outcome assessments of emerging AD therapies, such as disease stage and its relation to the target pathology, proposed time-based effects of disease-modifying therapies, and the possible influence of individual demographic or genetic factors and neuropathologic heterogeneity on clinical outcomes. Finally, we recommend the consideration of these factors, as appropriate, in the design and/or data reporting of future trials of disease-modifying therapies in AD.

Our review and interpretation of CLARITY-AD trial outcomes is limited to publicly available data and analyses of the 18-month trial, as published by the study investigators, at the time of this manuscript.

### Clinical outcome measures in CLARITY-AD

We here review the primary and secondary clinical outcome assessments (COAs) of CLARITY-AD using the definitions proposed by the US FDA-National Institute of Health (NIH) *B*iomarkers, *E*ndpoint*s*, and other *T*ools (BEST) glossary [12] (Table 2).

**Table 2:** Definitions of COA types according to the FDA-NIH BEST glossary^a^

***Term***	***Definition***
**Clinical outcome assessment (COA)**	An assessment of a clinical outcome (i.e., an outcome that describes or reflects how an individual feels, functions or survives) that can be made through a report by a clinician, a patient, a non-clinician observer or through a performance-based assessment
***Types of COAs***	
**Clinician-reported outcome (ClinRO)**	A measurement based on a report that comes from a trained health-care professional after observation of a patient’s health condition
**Patient-reported outcome (PRO)**	A measurement based on a report that comes directly from the patient (i.e., study participant) about the status of the patient’s health condition without amendment or interpretation of the patient’s response by a clinician or any other observer
**Observer-reported outcome (ObsRO)**	A measurement based on a report of observable signs, events, or behaviors related to a patient’s health condition by someone other than the patient or a health professional
**Performance outcome (PerfO)**	A measurement based on standardized task(s) actively undertaken by a patient according to a set of instructions

^a^The US Food and Drug Administration (FDA) and National Institute of Health (NIH) constructed a glossary of *B*iomarkers, *E*ndpoint*s*, and other *T*ools (BEST) [12] in an effort to harmonize various terms used to describe clinical and biomarker-based outcome measures in basic and clinical research

#### Primary clinical outcome

The CDR-SB is a subjective clinician-reported outcome (ClinRO) measure that consists of a structured interview with the patient and caregiver and covers six domains: Memory, Orientation, Judgment and Problem Solving, Community Affairs, Home and Hobbies, and Personal Care [3–5]. Each domain is rated from 0 to 3 (possible scores are 0, 0.5, 1, 2, or 3 for all domains except Personal Care which is given a score of 0, 1, 2, or 3). The CDR-SB score is generated by adding the scores of all 6 domains and ranges from 0 to 18 (0 indicating no impairment and higher scores indicating more severe impairment). The CDR-SB is frequently used as an outcome measure in AD clinical trials due to several advantages; it is a quantitative interval measure which is easy to compute, it closely tracks with clinical disease progression, it has been validated across different cohorts, and it combines both cognitive and functional domains into one overall score, so that CDR-SB scores provide a useful assessment of the impact of cognitive impairment on daily functions [13, 14]. The CDR-SB should not be confused with the global CDR score, which derives a single score (0, 0.5, 1, 2, or 3) from all domains using a scoring algorithm and is an ordinal measure of dementia severity. The mean baseline CDR-SB score in each arm of CLARITY-AD was ~3.2. At 18 months, the adjusted mean increase in the CDR-SB score from baseline in the treatment arm was 0.45 points less than that of the placebo arm (*p* < 0.001), reflecting 27% slowing of progression with lecanemab (Table 1).

#### Secondary clinical outcomes

##### ADCS-MCI-ADL

The ADCS-MCI-ADL is a subjective study partner-completed observer-reported outcome (ObsRO) measure of daily living activities in the prior 4 weeks which is frequently used as a functional outcome measure in clinical trials of early symptomatic AD [8]. Test scores are based on caregivers’ responses to 18 questions^2^ in a structured interview regarding the patient’s ability to perform various daily activities either independently, with supervision, or with physical assistance. Scores range from 0-53 with lower scores indicating greater impairment. At 18 months, the adjusted mean decline in ADCS-MCI-ADL scores was 2 points less with lecanemab compared to placebo (*p* < 0.001), reflecting 37% slowing of clinical progression (Table 1).

##### ADAS-Cog14

The ADAS-Cog14 is a hybrid performance outcome (PerfO) and subjective clinician-reported outcome (ClinRO) measure that covers 14 cognitive domains (range 0–90; higher scores indicating more severe impairment) [6]. The adjusted mean between-group difference (i.e., in the treatment arm compared to placebo) in the mean change from baseline to 18 months on ADAS-Cog14 was − 1.44 (*p* < 0.001), reflecting 26% slowing of progression with lecanemab (Table 1).

##### ADCOMS

ADCOMS (range 0–1.97; higher scores indicate more severe impairment) is a statistically derived composite measure which consists of 12 clinically sensitive items from other clinical scales; the 6 CDR domains, 4 items from ADAS-Cog, and 2 items from the Mini-Mental State Examination (MMSE) [7, 15]. This composite consists of cognitive and functional components of variable statistical weights [7]; scores typically range from 0.11 to < 0.31 in MCI and 0.31 to 0.77 in mild AD dementia [16]. Lecanemab slowed the worsening (i.e., increase) in baseline ADCOMS scores by 0.05 points (*p* < 0.001) at 18 months compared to placebo, reflecting 24% slowing of progression (Table 1).

##### Adverse events

Amyloid-related imaging abnormalities with brain edema/effusions (ARIA-E; lecanemab, 12.6%; placebo, 1.7%) or hemorrhage (ARIA-H; lecanemab, 17.3%; placebo, 9.0%) (Table 3), and infusion reactions (lecanemab, 26.4%; placebo, 7.4%) were reported in the 18-month trial [1]. Most ARIAs were asymptomatic; however, symptomatic ARIA-E or ARIA-H occurred at a frequency of 2.8% and 0.7%, respectively, in the lecanemab group. Brain atrophy (of undetermined clinical significance) has also been observed with lecanemab [17, 18]. At the time of this manuscript, three lecanemab-related deaths have been reported in the phase 3 open-label extension (OLE) study [19] (Table 3). (See [1] for a complete list of adverse events reported in CLARITY-AD).

**Table 3:** Incidence of ARIAs in relation to the *APOE4* genotype in CLARITY-AD^a^

ARIA adverse event^b^	Lecanemab	Placebo
***ARIA-E, n (%)***	113 (12.6%)	15 (1.7%)
*APOE4 non-carriers*	15 (5.4%)	1 (0.3%)
*APOE4 heterozygotes*	52 (10.9%)	9 (1.9%)
*APOE4 homozygotes*	46 (32.6%)	5 (3.8%)
***Symptomatic ARIA-E, n (%)***	25 (2.8%)	0%
*APOE4 non-carriers*	4 (1.4%)	0%
*APOE4 heterozygotes*	8 (1.7%)	0%
*APOE4 homozygotes*	13 (9.2%)	0%
***Serious Event with ARIA-E, n (%)***	7 (0.8%)	0%
*APOE4 non-carriers*	2 (0.7%)	0%
*APOE4 heterozygotes*	2 (0.4%)	0%
*APOE4 homozygotes*	3 (2.1%)	0%
***ARIA-H, n (%)***	155 (17.3%)	81 (9.0%)
*APOE4 non-carriers*	33 (11.9%)	12 (4.2%)
*APOE4 heterozygotes*	67 (14.0%)	41 (8.6%)
*APOE4 homozygotes*	55 (39.0%)	28 (21.1%)
***Symptomatic ARIA-H, n (%)***	6 (0.7%)	2 (0.2%)
*APOE4 non-carriers*	1 (0.4%)	0%
*APOE4 heterozygotes*	5 (1.0%)	1 (0.2%)
*APOE4 homozygotes*	0%	1 (0.8%)
***Serious Event with ARIA-H, n (%)***	5 (0.6%)	1 (0.1%)
*APOE4 non-carriers*	2 (0.7%)	1 (0.3%)
*APOE4 heterozygotes*	1 (0.2%)	0%
*APOE4 homozygotes*	2 (1.4%)	0%
***Isolated ARIA-H (no concurrent ARIA-E), n (%)***	80 (8.9%)	70 (7.8%)
*APOE4 non-carriers*	23 (8.3%)	11 (3.8%)
*APOE4 heterozygotes*	40 (8.4%)	35 (7.3%)
*APOE4 homozygotes*	17 (12.1%)	24 (18.0%)

*ARIA* amyloid-related imaging abnormalities, *ARIA-E* ARIA with edema/effusions, *ARIA-H* ARIA with hemorrhage, *APOE4* E4 allele of the apolipoprotein E gene

^a^Percentages are calculated based on the total number of participants in each corresponding cohort. Participants in the lecanemab arm (*n* = 898) were classified as *APOE4* non-carriers (*n* = 278), *APOE4* heterozygotes (*n* = 479), or *APOE4* homozygotes (*n* = 141). Participants in the placebo arm (*n* = 897) were classified as *APOE4* non-carriers (*n* = 286), *APOE4* heterozygotes (*n* = 478), or *APOE4* homozygotes (*n* = 133) (adapted from Cummings et al. [19])

^b^None of the deaths reported in the CLARITY-AD 18-month study (*n* = 6 and 7 in the lecanemab and placebo arms, respectively) were attributed to lecanemab or ARIAs. However, three lecanemab-related deaths have been reported in the open-label extension study; one in an *APOE4* noncarrier who was on anticoagulation and developed a macro-hemorrhage (ARIA-H), a second in an *APOE4* homozygote with cerebral amyloid angiopathy (CAA) who received tPA for a large vessel occlusion, and a third in an *APOE4* homozygote who developed severe ARIA-E and ARIA-H and a clinical syndrome resembling CAA-related inflammation

#### Understanding group level differences in CLARITY-AD outcomes

Thresholds for clinically meaningful group-level differences in CLARITY-AD clinical outcome measures have not been established. However, we here present our interpretation of the potential clinical relevance of group-level differences in each of the CLARITY-AD clinical outcome measures.

##### CDR-SB

As indicated by the verbal descriptors for the CDR-SB domains [3], slowing of CDR-SB worsening by 0.5 points in these early stages is expected to have a meaningful impact on daily functions and independence. For example, a 0.5-point difference in the Memory domain can mean the difference between “consistent slight or benign forgetfulness; partial recollection of events” and “moderate memory loss, more marked for recent events, which interferes with daily activities”. Similarly, a 0.5-point change in these early stages may distinguish “slight impairment in problem solving” *vs* “moderate difficulty in handling problems,” “slight impairment in community affairs” *vs* being “unable to function independently in these activities,” or “life at home, hobbies, and intellectual interests are slightly impaired” *vs* “mild but definite impairment of function at home, with more difficult tasks or more complicated hobbies and interests abandoned”. In all of these scenarios, a 0.5-point increase in CDR-SB scores likely denotes some loss of functional independence. While the full impact of slowing progression by 0.5 CDR-SB points over 18 months may vary across individuals based on what domains drive their total scores, at face value, many AD experts agree it is likely to be tangible and clinically important to patients and caregivers [9, 20].

The primary clinical outcome of CLARITY-AD was the mean difference between the treatment and placebo groups in the mean change from baseline CDR-SB scores at 18 months after adjusting for covariates (i.e., the adjusted mean “group-level” difference). As expected, this mean difference in the mean CDR-SB change from baseline of ~ − 0.5 points at 18 months, compared to placebo, was not necessarily observed in all participants in the treatment arm. Based on these results, and due to natural variation in participants’ response to treatment, it is expected that many participants treated with lecanemab achieved ≥ 0.5-point slowing in the CDR-SB change from baseline at 18 months compared to placebo, and therefore, likely had clinically noticeable benefits, while others did not. Further, as indicated by the 95% confidence interval for the adjusted mean group-level difference in mean CDR-SB change from baseline (− 0.67 to − 0.23, which reflects a range of possible values for the true mean)^3^, there is a possibility that the true value of this group-level mean difference was < ~0.5 points in size, in which case it is not likely to be clinically significant.

When interpreting treatment effects using the mean change in baseline CDR-SB as a clinical outcome, it is important to note that the CDR-SB scale increases by increments of ≥ 0.5 points with clinical progression (≥ 0.5 points in the early stages and ≥ 1 points in the advanced stages). A CDR-SB change of at least 0.5 points is required for a clinically noticeable effect in the early stages. However, the potential clinical significance of a 0.5-point change is dependent on disease stage; CLARITY-AD participants had early symptomatic AD in which the CDR-SB scores fall in the lower range of 0.5–6, where a 0.5-point change is likely to be noticeable. Conversely, in patients with more severe impairment, the CDR-SB typically increases by increments of ≥ 1, in which case a 0.5-point change is not likely to be associated with meaningful changes in cognition or functional abilities.

##### ADCS-MCI-ADL

A 1-point change in the ADCS-MCI-ADL can distinguish between a patient’s ability to perform certain daily tasks (e.g., balancing a checkbook, getting dressed, doing house chores such as cleaning, or performing a hobby) independently or only with supervision, and a 2-point difference would distinguish a patient’s ability to perform any of these activities independently *vs* the need for physical assistance. For other activities on this scale (e.g., using household appliances), a 2-point, but not a 1-point, difference may be clinically important. Overall, considering the adjusted mean and 95% CI (1.2 to 2.8) for the between-group difference in the mean ADCS-MCI-ADL change from baseline, lecanemab was likely associated with clinically significant slowing of functional decline on the ADCS-MCI-ADL at 18 months compared to placebo.

##### ADAS-Cog14

As ADAS-Cog14 measures cognitive, and not functional, performance, understanding the clinical meaningfulness of ADAS-Cog14 change requires associating this to a global or functional outcome. For context, individuals with early symptomatic AD who have clinically significant worsening over a 1-year follow-up have mean ADAS-Cog score (i.e., within-individual) changes of 2–5 points [21–24]. Thresholds for clinically meaningful group-level differences in ADAS-Cog14 change have not yet been established; however, considering the above findings, it is not likely that the relatively small group-level effect of lecanemab (− 1.44) on ADAS-Cog14 change in the 18-month trial was clinically significant.

##### ADCOMS

There is limited data in the literature to guide our interpretation of the clinical relevance of group-level differences in ADCOMS scores; however, based on our understanding of this composite measure, slowing of worsening in baseline ADCOMS scores by 0.05 points at 18 months with lecanemab, at face value, is not likely to be clinically meaningful.

In summary, lecanemab’s effects on changes in ADCS-MCI-ADL, and possibly the CDR-SB, but not ADAS-Cog14 or ADCOMS, scores at the end of the 18-month trial were likely clinically significant at the group level. At this juncture, as clinicians, we should ask these important questions: How can we reliably and objectively evaluate clinical meaningfulness of group-level differences in study outcomes? Further, how can study results reported as group-level differences be translated at the individual level?

### Clinical meaningfulness at the group and individual level

Most AD clinical trials, which employ a parallel-group design, report the statistical significance of between-group differences in clinical outcomes (e.g., mean group-level differences in mean change from baseline to endpoint on a validated scale) as a measure of treatment efficacy. As disease progression is slow in the early stages, even highly effective therapies may be associated with relatively small treatment effects; therefore, clinical trials generally require large cohorts to be adequately powered to detect statistically significant group-level differences in these early stages. However, examining the clinical relevance of statistically significant group-level differences in AD trial outcomes is not necessarily straightforward and may be subjective. For example, when evaluating treatment efficacy based on the group-level difference in change from baseline (i.e., absolute point difference) for an outcome measure on a scale with a large range, small differences may be misinterpreted as “modest” effects, while differences of the same magnitude on scales with narrower ranges may be misinterpreted as “large” effects [25]. Further, evaluating treatment outcomes based on absolute point differences between treatment and placebo groups on a specific scale should be considered in the context of the “dynamic” range of that scale (i.e., the expected change over time for any specific cohort) which is often different from its full-score range [26].

Evaluating group-level differences in study outcomes using validated thresholds that represent the minimal clinically important difference “MCID”^4^ in a clinical outcome measure (e.g., change from baseline) between the treatment and placebo groups provides an objective method to evaluate clinical meaningfulness of trial results. MCID thresholds at the group level for most AD trial outcomes have not been established [1]. Without such a reference point, evaluating the clinical meaningfulness of small, yet statistically significant, between-group differences can be challenging. Thus, establishing group-level MCID thresholds for AD trial outcomes, that are appropriate for disease stage, using standardized methods, and applying these “a priori”-determined thresholds in trial design and the evaluation of clinical trial data will allow the objective interpretation of the clinical relevance of trial results [21, 27]. Further, this approach will facilitate comparison of treatment effects across different trials and provide useful guidance for clinicians in their decision-making discussions with patients and families.

Another important consideration in understanding clinical trial outcomes is distinguishing “between-group differences” from “within-individual change”. While measuring group-level differences in study outcomes is the correct statistical approach to estimate treatment effects in parallel-group AD trials, estimates of between-group differences, and their statistical significance, depend not only on changes observed within individuals, but also on several factors in trial design, including per-group sample size. Therefore, estimates of group-level differences do not provide direct information regarding likely treatment effects at the individual level. Notably, the US FDA recommends that clinical meaningfulness be based on “individual-level” benefit [24, 28, 29]. For AD disease-modifying treatments, this translates into reductions in within-individual disease progression with treatment compared to placebo. To achieve this, within-individual thresholds for what would be considered a “clinically meaningful change” (“CMC”), or a meaningful within-patient change “MWPC,” in AD trial outcomes (i.e., reflective of disease progression) need to be established and/or validated, using anchor-based methods which rely on a rater’s assessment of meaningful progression over time, or less preferably, distribution-based methods. Individual-level thresholds for what is considered “minimal” or “moderate” worsening have been proposed for several AD trial outcomes using measures of global change (e.g., the Global Deterioration Scale [GDS] and the MCI- Clinical Global Impression of Change [MCI-CGIC]) as anchors in a large cohort with a clinical diagnosis of MCI [24] (Table 4). Anchor-based approaches can be applied to derive “CMC” (i.e., “MWPC”) thresholds in biomarker-confirmed cohorts with MCI, or mild dementia, due to AD [30]. It is important to note that, according to FDA and AD expert recommendations, individual-level “CMC” thresholds are not intended to define clinical meaningfulness of between-group differences reported in AD trials [20, 28], and vice versa, as this may lead to “misguided,” and perhaps unrealistic, expectations of emerging therapy outcomes.

**Table 4:** Proposed thresholds for clinically meaningful within-individual change in MCI^a^

***Outcome measure***	***Global anchor***	***Time interval, months***	***Threshold for minimal meaningful change***^b^	***Threshold for moderate meaningful change***^b^
*CDR-SB*	GDS	12	1.00–1.08	2.75–3.39
*CDR-SB*	MCI-CGIC	12	0.50–0.64	2.00–2.35
*ADAS-Cog11*	MCI-CGIC	12	2	3–4
*ADAS-Cog13*	MCI-CGIC	12	2	4–5
*MMSE*	GDS	36	2–3	6–7

*CDR-SB* Clinical Dementia Rating-Sum of Boxes, *GDS* Global Deterioration Scale, *MCI-CGIC* Mild Cognitive Impairment-Clinical Global Impression of Change, *ADAS-Cog11* 11-item cognitive subscale of the Alzheimer Disease Assessment Scale, *ADAS-Cog13* 13-item cognitive subscale of the Alzheimer Disease Assessment Scale, *MMSE* Mini-Mental State Examination

^a^Values represent proposed thresholds, or a range of thresholds, for what would be considered “minimal” and “moderate” clinically meaningful within-individual change in several clinical outcomes (using the GDS and the MCI-CGIC as global anchors) at various durations of follow-up. These values are adapted from mean and median anchor-based estimates of within-individual meaningful change which were generated by Lansdall et al. [24] in a clinically diagnosed (i.e., no biomarker confirmation) cohort with mild cognitive impairment (MCI) due to AD. On the GDS, “minimal” and “moderate” clinically meaningful change correspond to a 1-point and 2-point worsening, respectively. These thresholds for within-individual change are not intended to evaluate group-level differences in trial outcomes

^b^Values represent points on the outcome measures

To reconcile these differences, and in accordance with FDA guidance which emphasizes patient-focused drug development [28], we propose that the contextualization of clinical meaningfulness of clinical trial outcomes, reported as between-group differences, can be facilitated by reporting complementary data analyses that estimate within-individual change, such as reporting the proportion of participants in each study group who achieved the within-individual “CMC” thresholds for progression (i.e., “minimal’ or “moderate” worsening) using rater assessments of global change as “anchor” points [24, 30], or those who progressed on the global CDR score from baseline, over the trial period. An example of this approach is demonstrated by the TRAILBLAZER-ALZ 2 Phase 3 trial of another anti-amyloid agent, donanemab, in early symptomatic AD [31]. In addition to reporting group-level differences in the primary and secondary study outcomes, the study investigators examined, in supplementary non-gated time-based analyses, whether individual participants in each of the treatment and placebo groups reached prespecified “MWPC” thresholds of clinically important progression during the trial period [31, 32]. Further, in pre-specified gated (i.e., multiplicity-adjusted) secondary analyses, TRAILBLAZER-ALZ 2 reported that among participants with low/medium baseline tau burden, donanemab was associated with a 39% lower risk of progression (hazard ratio [HR] of 0.61, *p* < 0.001) in the global CDR score over 18 months compared to placebo, and that 47% of participants who received donanemab remained stable on the CDR-SB at 12 months compared to 29% of those who received placebo (*p* < 0.001) [31]. Conversely, published results of individual-level analyses in CLARITY-AD are limited. Exploratory analyses in CLARITY-AD suggest possible numerical benefits of lecanemab in reducing the risk of progression in the global CDR score compared to placebo (HR, 0.69, reflecting a 31% lower risk of progression) during the trial; however, these were multiplicity- unadjusted analyses and thus, should be interpreted with caution [1].

**Fig. 1:**
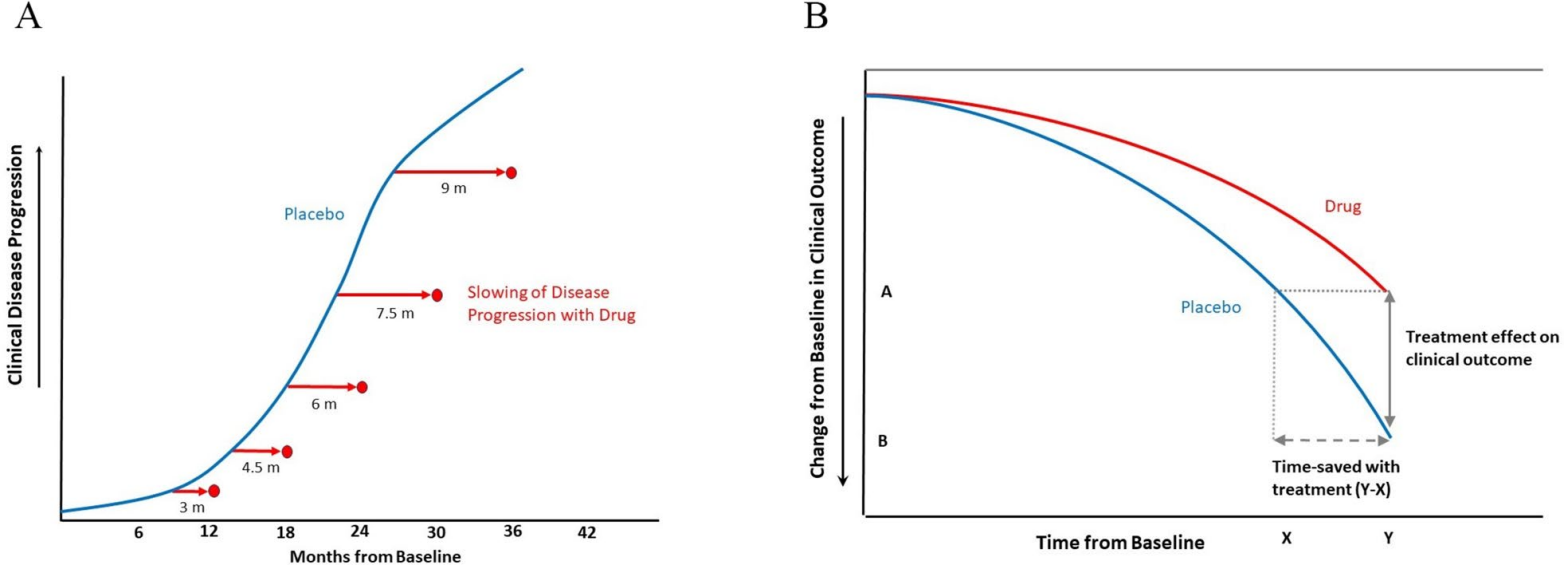
**A** Proposed time-based effects of disease-modifying treatments. In this example, a disease-modifying AD drug that slows clinical disease progression at a rate of 25% compared to placebo over the trial period is theoretically expected to slow (i.e., delay) clinical progression by approximately 3 months at 12 months, 4.5 months at 18 months, 6 months at 24 months, 7.5 months at 30 months, and 9 months at 36 months of treatment. Blue lines reflect clinical progression (i.e., increasing clinical severity) in the placebo group, and red dots reflect projected delays in progression with treatment at each study time-point. This model was adapted from progression models for repeated measures (Raket et al. [33]) and requires validation in clinical cohorts with long durations of follow-up. The blue line is only meant to illustrate a general example of nonlinear disease progression and is not meant to reflect any specific trajectory for disease progression or specific outcome measure. **B** Time-Saved as an illustration of disease-modifying effects. “Time-saved” reflects the difference in the time needed to reach a certain degree of worsening between the treatment and placebo groups (i.e., the duration of time by which cognitive or functional decline is delayed by treatment) and can be measured by time-component tests which account for non-linearity in disease progression. In this example, the rate of progression with treatment (red) is slower than that of placebo (i.e., natural disease progression) (blue). The treatment effect at any time-point reflects the difference in the clinical outcome between the treatment and placebo groups (e.g., difference between A and B for time-point Y). The decline in the clinical outcome at time-point Y in the treatment arm (reflected by A on the vertical axis) was reached at time-point X with placebo. Therefore, the difference between X and Y time-points reflects “time-saved” with treatment. Adapted from Dickson et al. [25]

For disease-modifying AD treatments, clinical benefits can also be communicated in terms of the proportional slowing of clinical disease progression. For example, lecanemab slowed clinical progression in CDR-SB by 27% compared to placebo [1] which is estimated to reflect slowing in CDR-SB progression by ~3.2 months at 12 months, ~5 months at 18 months, ~6.5 months at 2 years, and ~10 months at 3 years (Fig. 1A). As disease progression is nonlinear, progression models, which are based on continuous-time nonlinear mixed models for repeated measures, can estimate nonstandard treatment effects such as slowing or delaying disease progression over time (i.e., the “time-based’ treatment effect) [1, 33]. While these models may be useful to communicate projected benefits of disease-modifying treatments over time, they require validation in clinical cohorts with longer durations of treatment (i.e., beyond the 18-month trial period).

“Time-saved” is another useful metric which can be measured by time-component tests, using group- or individual-level data, and accounts for nonlinearity in disease progression or treatment response. “Time-saved” reflects the difference in the time needed to reach a certain degree of worsening between the treatment and placebo groups (i.e., the duration of time by which cognitive or functional decline is delayed with treatment), and has been applied to the interpretation of AD clinical trial data [25] (Fig. 1B). Reporting “time-saved” with a disease-modifying treatment may offer a more consistent and easily comprehendible metric to demonstrate clinical meaningfulness than absolute point or percentage differences on study outcomes, as the interpretation of the latter measures may be influenced by disease severity or treatment duration [26]. CLARITY-AD investigators performed a slope analysis using CDR-SB based on observed data and extrapolation to 30 months, which showed that participants who received lecanemab needed 25.5 months to reach the same level as placebo at 18 months, reflecting 7.5 months of “time-saved” with lecanemab [10].

It is also important to note that AD trial outcomes used to define clinical meaningfulness are mostly clinician-reported outcome (ClinRO) measures which may not fully capture, or closely correlate with, clinical benefits potentially perceived by patients and caregivers [30, 34]. A consortia-driven study identified the most important cognitive concerns reported by patients with amnestic MCI and their families (i.e., memory followed by orientation and language) and found that individual subcomponent tests from different statistically derived composite measures, many of which have been used as outcome measures in AD trials, aligned with each of these cognitive domains to variable extents [35]. Thus, incorporating patient- and caregiver-reported outcomes (i.e., PROs and ObsROs), as companion measures, into outcome assessments and data reporting in AD trials will allow for a multidimensional approach in evaluating drug efficacy and provide useful adjunct information to a larger audience of stakeholders [34]. Measuring PROs, that are validated for use in clinical trials and appropriate for disease stage, is encouraged by regulatory agencies as they capture patient experience data which can inform long-term evaluations of clinical meaningfulness and safety [34, 36, 37]. PROs will become particularly relevant as AD trials shift focus to earlier symptomatic stages when patient insight is preserved [34]. Efforts to develop and validate PROs that are appropriate for use in AD clinical trials are currently underway [38].

### Effects of time, disease stage, and neuropathologic complexity of AD

**Fig. 2:**
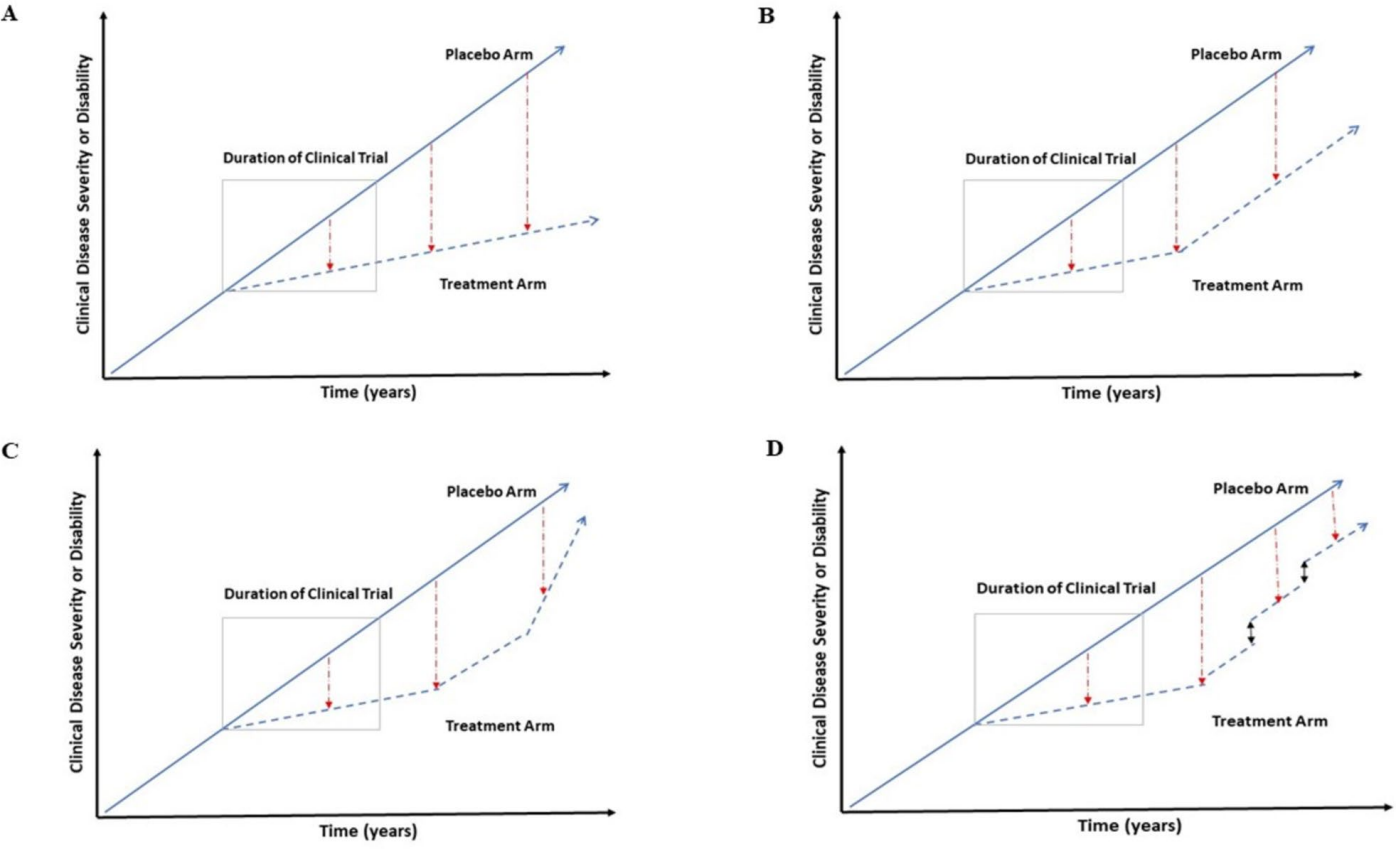
Examples of possible changes in drug-placebo differences with longer durations of disease-modifying treatments. **A** Drug-placebo differences increase with time. Nonlinear models of disease progression suggest that disease-modifying treatments, which reduce the rate of decline, theoretically result in increased drug-placebo differences with longer durations of treatment (adapted from Cummings et al. [39]). This model requires validation in clinical cohorts with long durations of follow-up (e.g., open-label extension studies and patient registries). Other possible scenarios include **B** drug-placebo differences plateau over time, so that the rates of decline in the drug arm become similar to those in the placebo arm with longer treatment durations. In this hypothetical scenario, further clinical benefits, as evidenced by further slowing of clinical decline, will halt after a certain duration of treatment (e.g., due to the target pathology being significantly reduced or removed); however, the largest absolute drug-placebo difference (as evidenced by new clinical baselines for the treatment arm) achieved with treatment may continue for a period of time. **C** Drug-placebo differences slowly diminish over time due to adverse events in the treatment arm. This hypothetical scenario is based on the notion that adverse events may increase over longer durations of treatment, potentially outweighing clinical benefits and leading to faster rates of progression in the treatment arm and reduced drug-placebo differences over time. **D** Drug-placebo differences slowly diminish over time due to gradual loss of clinical benefits in the treatment arm. This hypothetical scenario is an extension of **B** in which clinical benefits will plateau, leading to similar rates of decline in the treatment and placebo arm; however, previously attained benefits will gradually decline so that the drug-placebo differences will diminish over longer treatment durations and the clinical baselines of the two groups will converge. **A**–**D** are only examples of possible scenarios for how drug-placebo differences may change with longer treatment durations and are not meant to exhaustively outline all possible scenarios. Disease progression is shown as linear for simplicity

Considering that disease-modifying effects may not be fully evident over the relatively short durations of clinical trials, projected “theoretical” benefits (i.e., drug-placebo differences) over longer durations of treatment may be estimated using statistical models of disease progression and treatment response. Progression models suggest that time-based estimates of treatment effects for disease-modifying drugs are—theoretically—expected to increase over time [33, 39]. Preliminary (unadjusted) analyses of CLARITY-AD findings suggest that drug-placebo differences possibly increased over the 18-month trial duration for some clinical outcomes [1, 9]; however, it is important to note that data from long-term studies and patient registries is needed to determine whether the drug-placebo differences observed during the short trial duration would increase, plateau, or even potentially diminish if the drug is continued beyond 18 months. Figure 2 shows possible (i.e., hypothetical) scenarios for how drug-placebo differences may change over longer durations of a disease-modifying treatment in which (A) drug-placebo differences may potentially increase over time due to accrued drug benefits, (B) plateau over time as “maximum” clinical benefits, evidenced by slowing of clinical decline, are reached after the target pathology is removed or significantly reduced, or diminish over time due to (C) increased adverse events or (D) the gradual loss of previously attained benefits over longer durations of treatment. When estimating projected treatment benefits over time for disease-modifying therapies, it is also worthwhile to consider that, in contrast to delaying disease progression in early symptomatic stages in which independence and daily functions are relatively preserved, delaying disease progression in advanced symptomatic stages in which significant functional decline and loss of independence have already occurred, may possibly become less tangible or clinically relevant to patients and families.

Further, evaluating the clinical significance of an investigational treatment outcome should consider the time needed to achieve this outcome in the context of the drug’s mechanism of action [20]. For example, a symptomatic treatment that improves performance by 0.5 CDR-SB points at 6 months in the early stages will likely result in noticeable benefits, while potential benefits of a disease-modifying treatment that slows clinical worsening by 0.5 CDR-SB points at 18 months are much more difficult to discern. In contrast to symptomatic treatments whose pharmacological effects are expected to begin within hours or days, the disease-modifying process is much slower and can take several months or even years [20]. Although effective amyloid reduction with lecanemab was observed early in the trial, reduction of mean amyloid load to below positivity levels (~25–30 centiloids) in most participants was not achieved until 12–18 months of treatment [40], thus, the clinical benefits of near “complete” amyloid removal may have been delayed, and may not have been fully evident by the 18-month timepoint. CSF and plasma biomarker analyses in CLARITY-AD suggest possible benefits of lecanemab on biomarker measures of tau pathology, synaptic injury, and neuroinflammation during the 18-month trial [1]. While this data is promising, the duration for which any clinical benefits, or biomarker-based evidence, of potential disease-modifying effects would continue with longer durations of treatment, or following drug discontinuation, is yet to be determined^5^.

Disease stage and the neuropathologic complexity of AD are other important considerations in evaluating AD trial outcomes. Prior studies converge on the notion that amyloid is an early pathology that has nearly plateaued by the time symptoms appear and is not closely associated with cognition in early symptomatic AD [41, 42]. Therefore, anti-amyloid agents may have a better chance of success in earlier preclinical stages. Further, AD is a neuropathologically complex disease which, in addition to amyloid and tau, includes other co-pathologies, such as synuclein, TDP-43, and cerebrovascular disease, that independently and synergistically contribute to cognitive impairment [43, 44]. Therefore, it is not reasonable to expect that any disease-modifying treatment that only targets one pathology will result in large and directly evident clinical benefits over short trial durations. Rather, a combination of complementary therapies that target different pathologies will likely be needed for effective disease modification.

A significant degree of neuropathologic heterogeneity exists among participants within the same clinical disease stage, regarding the presence and severity of AD co-pathologies which—collectively—account for >50% of variance in cognitive outcomes [43, 44]. Emerging data from other AD trials suggests that clinical outcomes of anti-amyloid therapies in early symptomatic AD may be influenced by differences in baseline burden of tau pathology [9, 31, 45]. Further, post-hoc subgroup analyses from CLARITY-AD suggest that treatment outcomes may vary by individual demographic factors such as age, sex, race/ethnicity, and the *APOE4* genotype [1]. Although no definitive conclusions can be drawn from many of these analyses (e.g., those that are not based on randomization strata or involve small subgroups), observations from this study [1] and others [31], suggest that the potential influence of individual factors on clinical outcomes requires further investigation. Therefore, it will be important to consider the influence of baseline tau burden, and possibly other AD co-pathologies, and individual demographic or genetic factors in the design of future trials of anti-amyloid therapies. Studies which recruit a large number of participants from various under-represented populations and implement “a priori”-specified subgroup analyses with sample size justifications will further inform the potential contribution of individual factors to variations in treatment outcomes associated with emerging AD therapies.

Importantly, clinical decision-making involves evaluating not only a drug’s potential efficacy, but also its safety and potential side effects. Risk-benefit assessments of lecanemab in the clinic should include a careful review of the patient’s comorbidities and medication use, especially the use of anticoagulants which increase the risk for ARIA-H, and the *APOE4* genotype, which increases the risk for ARIA-E, ARIA-H, symptomatic, and recurrent ARIA [19] (Table 3). Understanding the potential influence of individual factors on treatment response and risk of adverse events is particularly important for *APOE4* homozygotes who appear to have a higher risk for ARIAs (Table 3), and possibly lower benefits, associated with treatment compared to *APOE4* noncarriers or heterozygotes in the 18-month trial.

Potential long-term benefits of lecanemab, when administered for longer than 18 months, should be carefully weighed against the risk for potentially more frequent or severe adverse events, including ARIAs. At this time, it remains unknown how the incidence or severity of ARIAs, or other adverse events, may change with longer durations of treatment, and what duration of treatment would have the most favorable risk-benefit ratio. Data from cohorts with longer durations of treatment (e.g., long-term extension studies and patient registries) is needed to address these knowledge gaps and guide risk-benefit assessments for our clinic populations. For context, a meta-analysis of the orally administered symptomatic cholinesterase inhibitor, donepezil, in mild to moderate AD suggests that it reduces clinical worsening in CDR-SB by 0.53 points at 6 months [46]; other studies have demonstrated its long-term safety and tolerability [47]. Therefore, it will be important to show that clinical benefits of emerging disease-modifying therapies, such as lecanemab, outweigh their potential risks in the long term and offer practical alternatives, or additions, to currently available symptomatic therapies which have more favorable, and well-established, safety profiles.

Finally, we emphasize that data from clinical cohorts in long-term extension studies and patient registries will be important to evaluate the long-term safety and efficacy of lecanemab in early symptomatic AD.

## Conclusion

CLARITY-AD demonstrated significant reductions in brain amyloid with lecanemab, which was associated with statistically significant differences in the primary (mean change in CDR-SB) and secondary (mean change in ADCS-MCI-ADL, ADCOMS, and ADAS-Cog14) clinical outcomes in the treatment group compared to placebo at the end of the 18-month trial. However, only group-level differences in ADCS-MCI-ADL, and possibly the CDR-SB—but not the other clinical outcome measures—were likely clinically meaningful at the 18-month timepoint. As group-level differences may be influenced by several factors in trial design and characteristics of the outcome measure being examined, and potentially open to various interpretations, the objective evaluation of clinical meaningfulness of future trial outcomes will be facilitated by the validation of stage-appropriate thresholds for group-level differences in the main clinical outcome measures employed by AD clinical trials.

While reporting group-level differences is the appropriate statistical approach to estimate treatment outcomes in parallel-group AD trials, adjunctive individual-level data summaries or analyses will facilitate the communication of trial results to patients, families, and various stakeholders. In accordance with FDA guidance which emphasizes the need to demonstrate individual-level benefit of emerging AD therapies, the contextualization of group-level outcomes can be achieved by reporting supplementary individual-level data analyses, such as those that measure the proportion of study participants who progressed during the study period using thresholds of clinically meaningful intraindividual change (i.e., using “CMC” or “MWPC” thresholds or progression to a higher global CDR score from baseline) in each of the study groups.

Treatment outcomes associated with disease-modifying therapies in AD clinical trials should be evaluated in the context of disease stage, treatment duration, and the neuropathologic complexity of AD. By the time individuals with AD become symptomatic, amyloid pathology has already plateaued and other AD pathologies such as tau contribute significantly to cognitive decline. Therefore, anti-amyloid agents may be more effective if administered in earlier preclinical stages, and outcome assessments of anti-amyloid agents in early symptomatic AD should account for the potential influence of other AD pathologies on study outcomes. Potential clinical effects of disease-modifying therapies, demonstrated by slowing of clinical progression, may not be fully evident during the relatively short durations of clinical trials. Progression models based on repeated measures propose that these benefits— theoretically—increase over time; however, these models require validation using long-term clinical data. At this time, whether and how the efficacy of lecanemab, or the frequency or severity of adverse events such as ARIAs, may change with longer durations of treatment (i.e., >18 months), remains unknown. We also discuss the potential influence of individual factors (e.g., demographic factors and *APOE4* genotype) on treatment response and/or risk for adverse events and advocate for the inclusion of these factors into the design of future clinical trials of disease-modifying AD therapies, which will guide clinicians regarding their appropriateness for use in various patient populations.

Finally, we emphasize the need for data from cohorts with longer durations of follow-up, such as those in long-term extension studies and patient registries, to evaluate the long-term efficacy and safety of lecanemab in early symptomatic AD and guide decision-making and risk-benefit assessments in the clinic.

## Data Availability

Not applicable

## Footnotes

1 CLARITY-AD analyses of the clinical outcomes used mixed models for repeated measures. The reported means are adjusted least-squares means which reflect the model estimates of the group-level means after adjusting for covariates

2 The ADCS-MCI-ADL is composed of 24 questions; however, only responses to questions 1-18 are included in the total score.

3 Interpretation of a 95% CI: if multiple samples of the same size were drawn from the same population and a 95% CI calculated for each sample, we would expect the true population mean to be found within 95% of these CIs.

4 The term MCID has been used inconsistently in the literature to either describe clinically meaningful group-level differences or within-individual change. In our discussion regarding clinical meaningfulness, we use “MCID” to describe group-level differences and “CMC” (or “MWPC”) to describe within-individual change.

5 Limited data from a small cohort in an open-label extension of a phase 2 study of lecanemab suggests that further slowing of decline in the treatment arm halted, but the absolute drug-placebo difference in clinical outcomes remained the same for at least ~2 years, following drug discontinuation.
